# Digoxin treatment reactivates *in vivo* radioactive iodide uptake and correlates with favorable clinical outcome in non‐medullary thyroid cancer

**DOI:** 10.1007/s13402-021-00588-y

**Published:** 2021-02-03

**Authors:** Thomas Crezee, Marika H. Tesselaar, James Nagarajah, Willem E. Corver, Johannes Morreau, Catrin Pritchard, Shioko Kimura, Josephina G. Kuiper, Ilse van Engen-van Grunsven, Jan W. A. Smit, Romana T. Netea-Maier, Theo S. Plantinga

**Affiliations:** 1 Department of Pathology, Radboud University Medical Center and Radboud Institute for Molecular Life Sciences, Geert Grooteplein Zuid 10, Nijmegen, 6500 HB The Netherlands; 2 Department of Radiology & Nuclear Medicine, Radboud University Medical Center and Radboud Institute for Molecular Life Sciences, Nijmegen, The Netherlands; 3 Department of Pathology, Leiden University Medical Center, Leiden, The Netherlands; 4 Department of Pathology, University of Leicester, Leicester, LEI7RH UK; 5 Laboratory of Metabolism, National Cancer Institute, National Institutes of Health, Bethesda, MD 20892 USA; 6 PHARMO Institute, Utrecht, The Netherlands; 7 Department of Internal Medicine, Radboud University Medical Center and Radboud Institute for Molecular Life Sciences, Nijmegen, The Netherlands

**Keywords:** Non‐medullary thyroid cancer, Digoxin, Radioactive iodide, Autophagy, Redifferentiation

## Abstract

**Purpose:**

Non-medullary thyroid cancer (NMTC) treatment is based on the ability of thyroid follicular cells to accumulate radioactive iodide (RAI). However, in a subset of NMTC patients tumor dedifferentiation occurs, leading to RAI resistance. Digoxin has been demonstrated to restore iodide uptake capacity *in vitro* in poorly differentiated and anaplastic NMTC cells, termed redifferentiation. The aim of the present study was to investigate the *in vivo* effects of digoxin in TPO-Cre/LSL-Braf^V600E^ mice and digoxin-treated NMTC patients.

**Methods:**

Mice with thyroid cancer were subjected to 3D ultrasound for monitoring tumor growth and ^124^I PET/CT for measurement of intratumoral iodide uptake. Post-mortem analyses on tumor tissues comprised gene expression profiling and measurement of intratumoral autophagy activity. Through PALGA (Dutch Pathology Registry), archived tumor material was obtained from 11 non-anaplastic NMTC patients who were using digoxin. Clinical characteristics and tumor material of these patients were compared to 11 matched control NMTC patients never treated with digoxin.

**Results:**

We found that in mice, tumor growth was inhibited and ^124^I accumulation was sustainably increased after short-course digoxin treatment. Post-mortem analyses revealed that digoxin treatment increased autophagy activity and enhanced expression of thyroid-specific genes in mouse tumors compared to vehicle-treated mice. Digoxin-treated NMTC patients exhibited significantly higher autophagy activity and a higher differentiation status as compared to matched control NMTC patients, and were associated with favourable clinical outcome.

**Conclusions:**

These *in vivo* data support the hypothesis that digoxin may represent a repositioned adjunctive treatment modality that suppresses tumor growth and improves RAI sensitivity in patients with RAI-refractory NMTC.

**Supplementary Information:**

The online version contains supplementary material available at 10.1007/s13402-021-00588-y.

## Introduction

Non-medullary thyroid cancer (NMTC) is the most prevalent endocrine tumor with an increasing incidence [1]. Thyroidectomy followed by eradication of primary tumor remnants and small metastases by radioactive iodide (RAI) treatment is a successful treatment regimen for the majority of NMTC patients, leading to high remission rates and long-term disease-free survival [2, 3]. However, 30–40 % of patients with metastatic NMTC develop RAI-refractory tumors. Since distant metastases in RAI-refractory NMTC patients are mostly located in lung and bone, these patients are often not eligible for curative surgery [4, 5]. Moreover, although some patients with RAI-refractory tumors can achieve long term survival by integrating multimodal therapeutic strategies including external beam radiotherapy, chemotherapy and targeted kinase inhibitor therapy, they rarely achieve curation. This further emphasizes the need for novel therapeutic options [6–9].

Recently, we have demonstrated that loss of autophagy activity is associated with NMTC dedifferentiation and RAI resistance, suggesting that modulation of autophagy may represent a target of adjunctive therapy to improve RAI responsiveness [10–12]. Accordingly, activation of autophagy for induction of NMTC redifferentiation *in vitro* was established by treatment with digoxin and other digitalis-like compound (DLC) analogues. DLCs are well tolerated Na^+^/K^+^ ATPase inhibitors that activate autophagy by increasing intracellular Ca^2+^ concentrations and inducing ERK, AMPK and TFEB signalling [13–15]. Interestingly, DLCs restore functional expression of the human sodium-iodide symporter (NIS), facilitating increased RAI uptake in dedifferentiated and anaplastic NMTC cells [16, 17].

Anticancer effects of digoxin have been studied in several other cancer types including prostate, lung, colon and breast cancer. These studies differ in their findings. Some studies indicate that digoxin use is not significantly associated with cancer incidence or cancer-specific mortality in a number of malignancies [18–21]. In contrast, other human studies suggest that digoxin could modify cancer initiation or progression in certain tissue types [22–24]. Potential effects of digoxin treatment on NMTC development, progression and treatment response have not been studied so far.

The aim of the present study is to validate previous *in vitro* findings in *in vivo* settings, in both a mouse model and in retrospectively collected human tumor specimens. TPO-Cre/LSL-Braf^V600E^ mice, that spontaneously develop RAI-resistant papillary thyroid cancer, were used to investigate the potential capacity of digoxin to restore RAI uptake *in vivo* and to elucidate the molecular mechanisms involved. Since digoxin is an established medication for heart disease, NMTC patients using digoxin for heart disease at the time of diagnosis and for initial treatment of NMTC, were retrospectively selected. Additional matched control NMTC patients were selected to enable investigations into the effects of digoxin treatment on intratumoral differentiation status, RAI treatment response and clinical outcome of NMTC patients.

## Materials and methods

### Mouse experiments

To generate mice that harbour thyroid-specific expression of endogenous Braf^V600E^, TPO-*Cre* mice (kindly provided by S. Kimura, National Cancer Institute, NIH, Bethesda, USA) were crossed with LSL-Braf^V600E^ mice (kindly provided by C. Pritchard, University of Leicester, UK), as described previously [25, 26]. At the age of 8–10 weeks both healthy wild-type and tumor-bearing Braf^V600E^ mutant mice were selected for further experiments. Male and female mice that harboured the Braf^V600E^ mutation were randomly divided into three groups: DMSO vehicle treatment (N = 3), treatment with digoxin 20 µg (N = 6) or treatment with digoxin 60 µg (N = 6). The digoxin dosages were based on previous mouse studies [27]. Wild-type mice were included to enable comparison with the physiological range of iodide uptake (N = 3). Before treatment initiation, baseline measurements of weight, tumor volume and iodide uptake were performed. Subsequently, approximately 3 MBq ^124^I was administered by oral gavage after which the mice were imaged by PET/CT (Siemens Inveon, Erlangen, Germany) 24 h and 72 h post-injection under inhalation of isoflurane anaesthesia. Next, vehicle or digoxin treatment was initiated, consisting of daily intraperitoneal injections with either 20 µg or 60 µg digoxin or DMSO (vehicle control). Subsequent ^124^I injections and PET/CT imaging were performed to assess post-treatment iodide uptake after one, two and three weeks of digoxin or vehicle treatment. PET/CT scan analyses were performed using Inveon software (Siemens, Erlangen, Germany). Regions of interest (ROI) were determined by setting the minimum threshold at 35,000 Bq/ml and 7500 Bq/ml for the 24 h and 72 h ^124^I scans, respectively. Mean Bq/ml uptake was corrected for tumor volume (mm^3^) and amount of administered ^124^I activity (MBq). Pre- and post-treatment thyroid tumor volumes were assessed by three-dimensional (3D) ultrasound imaging (Vevo2100, Fujifilm VisualSonics, Toronto, ON, Canada). Finally, mice were sacrificed and thyroid tumors were surgically removed. Of every mouse tumor one thyroid lobe was snap-frozen in liquid nitrogen for molecular analysis whereas the other lobe was fixed in 4 % formalin and paraffin embedded for immunohistochemistry. All animal studies were approved by the Animal Welfare Body of the Radboud University Medical Center, Nijmegen, The Netherlands and were in compliance with the NIH Guide for the Care and Use of Laboratory Animals.

### Selection of digoxin‐treated and matched control NMTC patients

NMTC patients were selected from the pathology database of the nationwide network and registry of histo- and cytopathology in The Netherlands (PALGA, Houten, The Netherlands) [28]. Patients diagnosed with anaplastic thyroid cancer were excluded. Subsequently, data on prescribed medication from the nationwide Out-patient Pharmacy Database of the PHARMO Database Network (PHARMO Institute, Utrecht, The Netherlands) were linked on a patient level to the obtained PALGA dataset by a validated algorithm [29, 30]. Ultimately, detailed clinical data available at the time of diagnosis of all NMTC patients diagnosed in The Netherlands between 1999 and 2017, including pathology reports and medication data, were gathered. From this nationwide search, a total of 11 NMTC patients were eligible after applying the following inclusion criteria: patients treated with digoxin at the time of diagnosis and initial treatment of NMTC. Total or near-total thyroidectomy was performed in all selected NMTC cases in addition to modified radical lymph node dissections in patients with confirmed nodal metastases. RAI ablation of residual thyroid tissue was considered 4–6 weeks after surgery. Patients were repeatedly treated with RAI to reach remission, if indicated. Remission was defined as undetectable TSH stimulated thyroglobulin (Tg) in the absence of anti-Tg antibodies and no evidence of loco-regional disease or distant metastasis on the whole-body iodine scans (WBS) and/or neck ultrasonographic examinations at 6–9 months after RAI ablation at the last follow-up visit. Tumor recurrence was defined as new evidence of loco-regional disease or distant metastasis after successful primary therapy. Persistent disease was defined as detectable Tg and/or evidence of loco-regional disease or distant metastases. To compare digoxin-treated NMTC patients with appropriately matched control patients, other NMTC patients never treated with digoxin were selected that matched for age, sex, histological tumor type, TNM staging at diagnosis and intratumoral mutation status. Data on co-medication comprised drugs that are classified as A10 (drugs used in diabetes), C01 (cardiac therapy), C02 (anti-hypertensives), C03 (diuretics), C04 (peripheral vasodilators), C05 (vasoprotectives), C07 (beta blocking agents), C08 (calcium channel blockers), C09 (agents acting on the renin-angiotensin-aldosterone system) or C10 (lipid modifying agents) medication according to the Anatomical Therapeutic Chemical (ATC) classification system. Patients treated with calcium channel blockers were excluded. Of all selected NMTC patients, archived formalin-fixed paraffin embedded (FFPE) primary tumor tissue specimens were collected for genetic, transcriptomic and protein analyses. Patient follow-up times ranged from 3 to 15 years after diagnosis. Information on clinical outcome was not part of the inclusion criteria, as this was gathered after the selection process. Collection, storage and use of archival tissue and patient data were performed in compliance with the “Code of Proper Secondary Use of Human Tissue in the Netherlands” (http://www.fmwv.nl and www.federa.org). This study was approved by the Institutional Review Board (IRB) under application CMO-2015-1762. This study followed the ethical guidelines of the IRB. The IRB waived the need for consent for use of the archived samples and the samples were analysed anonymously.

### DNA and RNA isolation from human FFPE tissues

For DNA isolation, 60 µm FFPE tissue slices were digested overnight at 56 °C in the presence of TET-lysis buffer (10 mM Tris/HCl pH 8.5, 1 mM EDTA pH 8.0, 0.01 % Tween-20), 5 % Chelex-100 (Bio-Rad, Hercules, CA, USA), 15 µg/ml GlycoBlue (Life Technologies, Carlsbad, CA, USA) and 400 µg proteinase K (Qiagen, Valencia, CA, USA), followed by inactivation at 95 °C for 10 min. For DNA isolation, the supernatant was transferred after centrifugation (16,000 rpm), cooled on ice and precipitated in the presence of 100 % EtOH and 1/33 volume 3 M NaAc (pH 5.2). Pellets were washed with cold 70 % EtOH and dissolved in 80 µl Tris-EDTA after which DNA concentrations were determined using a Qubit Broad Range Kit (Thermo Fisher, Waltham, MA, USA). For RNA isolation, 60 µm FFPE tissue slices were digested overnight at 56 °C in the presence of 240 µl lysis buffer (Qiagen, Valencia, CA, USA) and 400 µg proteinase K. Next, supernatants were transferred after centrifugation (16,000 rpm) and mixed with RNA-Bee. Subsequently, RNA was isolated by phase separation with chloroform and precipitated by isopropanol, according to the manufacturer’s instructions (Thermo Fisher, Waltham, MA, USA).

### Real‐time quantitative PCR

Snap-frozen murine thyroid tumor lobes were thawed and lysed in TRIzol reagent (Invitrogen), followed by total RNA purification performed according to the manufacturer’s instructions. Isolated RNA was transcribed into cDNA using an iScript cDNA synthesis kit (Bio-Rad, Hercules, CA, USA). Quantitative PCR was performed using the SYBR green method (Thermo Fisher, Waltham, MA, USA), with primers for the following mouse TDS genes: Dio1, Dio2, Duox1, Duox2, Foxe1 (Ttf2), Glis3, Nkx2-1 (Ttf1), Pax8, Slc26a4 (Pendrin), Slc5a5 (NIS), Slc5a8, Tg, Thra, Thrb, Tpo and Tshr. The primer sequences are listed in Table S1.

### Intratumoral mutation profiling

Somatic mutations in human NMTC tumor tissues were detected using our in-house Cancer Hotspot Panel based on single-molecule molecular inversion probes (smMIPs), as described previously [31]. Isolated DNA from FFPE tissues with a tumor cell percentage of > 60 % was subjected to library preparation after which clinically relevant regions were sequenced of the following genes: *AKT1, BIRC3, BRAF, CHEK2,CTNNB1, CXCR4, EGFR, ERBB2, EZH2, GNA11, GNAQ, GNAS, H3F3A, H3F3B, HRAS, IDH1, IDH2, JAK2, KIT, KRAS, MPL, MSH2, MYD88, NRAS, PDGFRA, PIK3CA, SF3B1, SLC7A8* and *ZNF2*.

### Gene fusion analysis

DNA/RNA was isolated from human punched tumor tissue with a tumor cell percentage of > 50 %. Gene fusion analysis was performed using Next Generation Sequencing (Archer FusionPlex CTL Panel) and data were analysed using Archer Analysis software (version 5). Relevant fusions of the following target genes were sequenced: ALK (5’; exons 2, 4, 6, 10, 16–23, intron 19), AXL (3’; exons 18–20), BRAF ( 5’; exons 7–11, 3’; exons 7, 8, 10), CCND1 (5’; exons 1–4, 3’; exons 1, 2, 4), FGFR1 (5’; exons 2, 8–10, 17, 3’; exon 17), FGFR2 (5’; exons 2, 5, 7–10, 3’; exon 17), FGFR3 (5’; exons 3, 5, 8–10, 3’; exon 17, intron 17), MET (5’; exons 2, 4–6, 13, 14, 16, 17, 21, 3’; exon 2), NRG1 (5’; exons 1, 2, 3, 6), NTRK1 (5’; exons 2, 4, 6, 8, 10–13), NTRK2 (5’; exons 5, 7, 9, 11–17), NTRK3 (5’; exons 4, 7, 10, 13–16), PPARG (5’; exons 1, 2, 3, 5), RAF1 (5’; exons 4–7, 9–12), RET (5’; exons 2, 4, 6, 8, 9–14), ROS1 (5’; exons 2, 4, 7, 31–37), THADA (3’; exons 24–30, 36, 37). In addition, the FusionPlex-CTL hotspot panel also detects mutations in BRAF (exon 11, 15), HRAS, NRAS (exon 2 and 3, codon 12, 13, 61), KRAS (exon 2, 3 and 4, codon 12, 13, 61 and 146) and the EGFRvIII variant.

### TERT promoter mutation analysis

TERT promoter mutations C228A, C228T (at position − 124 from translation start site) and C250T (-146 from translation start site) were detected by conventional PCR and Sanger sequencing. The *TERT* promoter region was amplified using the following M13-sequence extended primers: forward 5’-TGT-AAA-ACG-ACG-GCC-AGT-CCC-TTC-ACC-TTC-CAG-CTC-3’ and reverse 5’-CAG-GAA-ACA-GCT-ATG-ACC-AGC-GCT-GCC-TGA-AAC-TCG − 3’. DNA was amplified using AmpliTaq PCR 360 Gold Master Mix (Thermo Fisher, Waltham, MA, USA) and the following PCR program: 95 °C for 10 min followed by 95 °C for 30 s, 58 °C for 30 s and 72 °C for 1 min (38 cycles) and a final step of 72 °C for 7 min. Subsequently, Sanger sequencing was performed using M13 primers and TERT promoter mutations were called using Sanger chromatogram software (Sequencher 4.8, Gene Codes Corp., Ann Arbor, MI, USA).

### FFPE compatible RNA sequencing

Isolated RNA obtained from human FFPE tissues with a tumor cell percentage of > 80 % was processed for RNA sequencing by DNAse treatment, RNA demodification, RNA fragmentation (if necessary), first and second-strand cDNA synthesis and library preparation according to the Ovation SoLo RNA-Seq System (NuGEN, San Carlos, CA, USA). Subsequently, RNA sequencing was performed using Illumina NextSeq500. Raw unmapped RNA sequencing data were mapped on the UCSC hg19 human genome assembly and were analysed using Tuxedo tools TopHat and CuffDiff in the RNA sequencing module on the GenePattern server of the Broad Institute to assess differential expression. Processed data were visualized using the Rstudio CummeRbund tool [32]. Raw RNA sequencing data are deposited in the GEO database under accession number GSE112202.

### Immunofluorescent stainings of FFPE tissues

Human and murine FFPE tumor tissues were sectioned (4 µm) and mounted onto glass slides. Sections were deparaffinized in xylene and rehydrated in 100 %, 96 % and 70 % ethanol. Histological examination was performed based on haematoxylin-eosin stainings. Autofluorescence was blocked by Sudan B Black as reported before [33]. If applicable, antigen retrieval was performed by 10 min boiling in sodium citrate buffer (10 mM Sodium Citrate, 0.05 % Tween 20, pH 6.0). Non-specific binding sites were blocked with 20 % normal goat serum in PBS. Stainings for the proliferation marker Ki-67 were performed using a monoclonal primary rabbit-anti-mouse/human antibody (clone SP6, Abcam, Cambridge, UK) diluted 1:200 in PBS/1 % BSA. For detection of the autophagy marker LC3 and the transcription factor FOS polyclonal primary rabbit-anti-mouse/human antibodies (LC3: ab48394, FOS: ab209794, both from Abcam, Cambridge, UK) diluted 1:200 in PBS/1 % BSA were used. After overnight incubation with primary antibody at 4 °C, tissue sections were washed with PBS and incubated for one hour at room temperature with a secondary goat anti-rabbit Alexa Fluor 647 antibody (Thermo Fisher, Waltham, MA, USA) diluted 1:100 in PBS/1 % BSA. Finally, tissue sections were mounted with Fluoromount-G containing DAPI (Southern Biotech, Birmingham, AL, USA). For LC3 immunofluorescence, the antigen retrieval step was omitted. Negative controls were processed with the primary antibody omitted. Staining images were acquired in a blinded fashion using fluorescent microscopy (Leica DMI6000B, Leica Microsystems, Wetzlar, Germany). For quantitative assessment, the percentages of Ki-67 and FOS positive nuclei and LC3-II positive puncta per 100 cells were quantified using FIJI software. Scoring results were generated at least in quintuple for each tissue section.

### Statistical analysis

Statistical significance was tested using Student’s T test, Mann Whitney *U* test, Spearman’s rank correlation coefficient, Wilcoxon matched-pairs signed rank test or by repeated ANCOVA measures with baseline correction, when appropriate. *P*-values < 0.05 were considered statistically significant. All experiments were performed by incorporating at least 3 technical replicates. For RNA sequencing data a false discovery rate of 0.05 was incorporated. All statistical tests were performed using GraphPad Prism 5.0.

## Results

### Transgenic TPO-Cre/LSL-Braf^V600E^ mouse studies

#### Digoxin inhibits tumor growth and improves iodide uptake in Braf^V600E^ mouse NMTCs

**Fig. 1:**
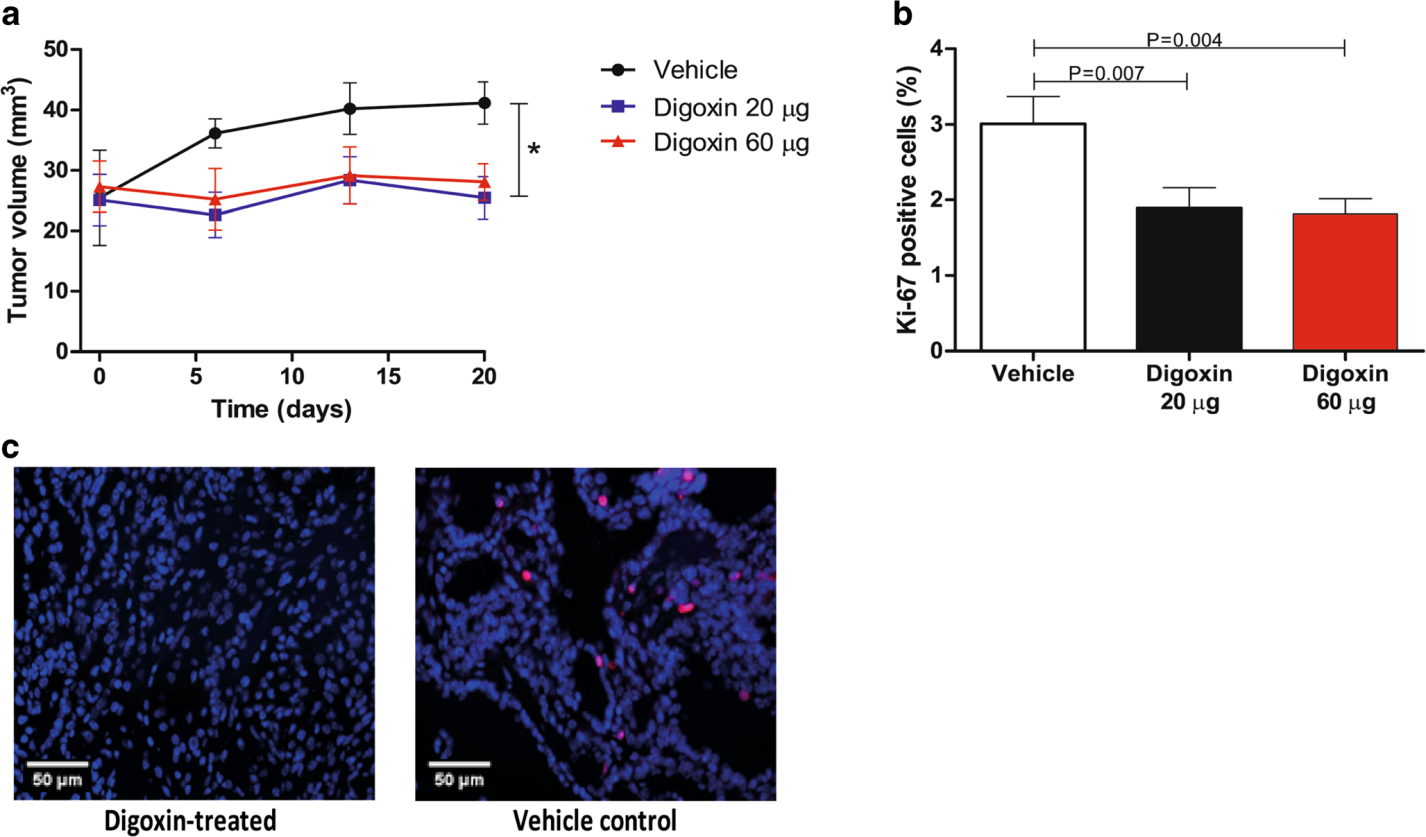
Digoxin treatment inhibits growth of Braf^V600E^ mouse tumors.** a** Tumor volumes in time in TPO-Cre/LSL-Braf^V600E^ transgenic mice treated with either DMSO vehicle (N = 3), 20 µg digoxin (N = 6) or 60 µg digoxin daily (N = 6). Data are means ± SD, *P < 0.05 (repeated measures ANCOVA with baseline correction). **b** Percentages of Ki-67 positive cells in post-mortem tumor tissue of TPO-Cre/LSL-Braf^V600E^ transgenic mice treated with either DMSO vehicle (N = 3), 20 µg digoxin (N = 6) or 60 µg digoxin daily (N = 6). **c** Representative immunofluorescent images of Ki-67 expression in digoxin-treated (left) and vehicle control (right) murine tumors. Scoring results were generated in quintuple for each tissue section. Data are means ± SEM, *P < 0.05 (Mann-Whitney *U* test)

**Fig. 2:**
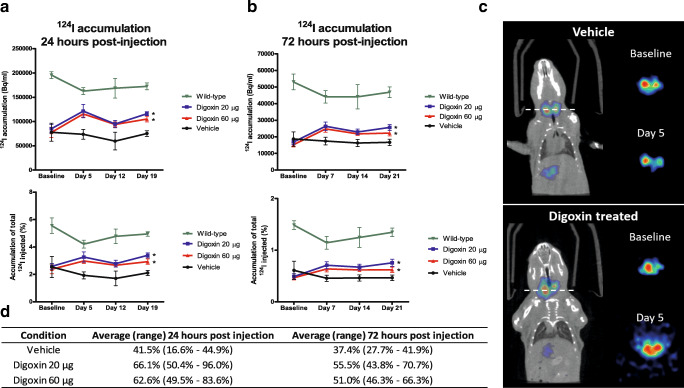
Digoxin treatment enhances ^124^I accumulation in Braf^V600E^ mouse tumors. Intratumoral ^124^I accumulation in TPO-Cre/LSL-Braf^V600E^ transgenic mice treated with either DMSO vehicle (N = 3), 20 µg digoxin (N = 6) or 60 µg digoxin daily (N = 6) at baseline and at three time points after initiation of digoxin treatment. PET/CT scans were performed either 24 hours (**a**) or 72 hours (**b**) after ^124^I injection at baseline and day 5, day 12, day 19 of digoxin treatment. Data are expressed as measured intratumoral ^124^I activity (Bq/ml, upper panels) and as intratumoral percentage of injected ^124^I activity (lower panels). Data are means ± SEM, *P < 0.05 (repeated measures ANCOVA with baseline correction). **c** Representative frontal PET/CT images of one vehicle and one digoxin treated mouse; dotted line represents the axial plane. Axial images are shown for ^124^I uptake at baseline and day 5 after vehicle or digoxin treatment. Images were corrected for total injected ^124^I activity. **d** Relative percentages (average and range) of ^124^I accumulation 24 and 72 hours after ^124^I injection in vehicle and digoxin-treated mice in relation to physiological ^124^I uptake in thyroids of wild-type mice (100 % reference)

TPO-Cre/LSL-Braf^V600E^ transgenic mice were studied to assess the effects of digoxin treatment on the proliferation and differentiation of Braf^V600E^-driven thyroid tumors. For tumor growth measurements, 3D ultrasound was performed at baseline and at day 5, 12 and 19 after the start of treatment with either vehicle, 20 µg digoxin or 60 µg digoxin. Tumor volumes at baseline were similar between treatment groups. During treatment, marked differences in tumor volume were observed. The tumor volumes of both 20 µg and 60 µg digoxin-treated mice remained unchanged over time, whereas in vehicle-treated mice tumor growth continued (Fig. 1a). Accordingly, immunofluorescent staining for the proliferation index marker Ki-67 on post-mortem tumor tissues revealed that the percentage of Ki-67 positive tumor cells decreased after treatment with digoxin compared to vehicle-treated mice (Fig. 1b, c). For these parameters, no digoxin dose-dependent effects were apparent. Upon haematoxylin-eosin staining, no morphological differences were observed between the tumors from digoxin-treated and vehicle-treated mice (data not shown). Furthermore, ^124^I PET/CT scanning before and after 5, 12 and 19 days of digoxin treatment revealed increased ^124^I accumulation in thyroid tumors at 24 h and 72 h after ^124^I injection in mice treated with either 20 µg or 60 µg digoxin (60 % and 43 % increases compared to baseline, respectively). No increased ^124^I uptake was observed in thyroid tumors of vehicle-treated mice. These differences in ^124^I uptake were already observed after 5 days of digoxin treatment and were retained at day 12 and day 19. Compared to physiological ^124^I uptake in thyroids of wild-type mice as 100 % reference, the relative percentage of iodide uptake 24 h and 72 h after ^124^I injection was increased in tumors of mice treated with either 20 µg or 60 µg digoxin as compared to vehicle treated mice (Fig. 2a, b, d). Representative PET/CT images are shown in Fig. 2c.

#### Digoxin enhances autophagy activity and thyroid‐specific gene expression in Braf^V600E^ mouse tumors

**Fig. 3:**
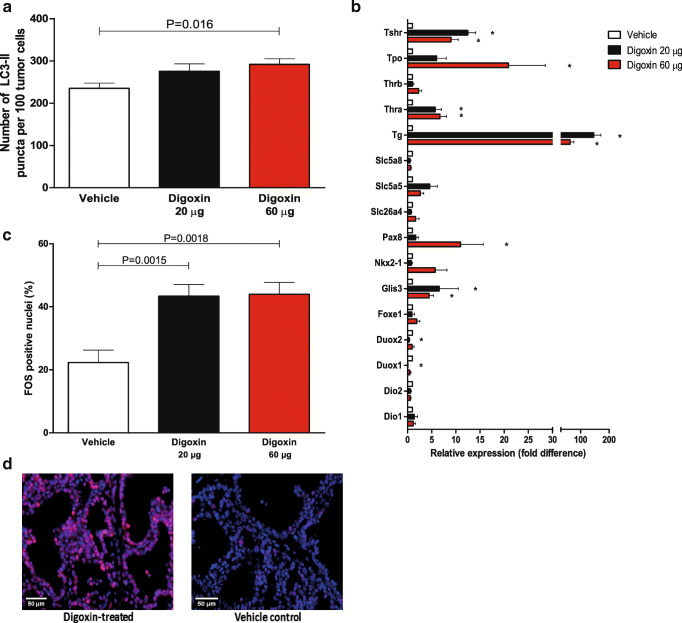
Digoxin treatment enhances autophagy activity, thyroid-specific gene expression and nuclear FOS levels in Braf^V600E^ mouse tumors.** a** Quantification of the number of LC3-II positive puncta per 100 tumor cells in post-mortem tumor tissue of TPO-Cre/LSL-Braf^V600E^ transgenic mice treated with either DMSO vehicle (N = 3), 20 µg digoxin (N = 6) or 60 µg digoxin daily (N = 6). Scoring results were generated in quintuple for each tissue section. Data are means ± SEM, *P < 0.05 (Mann-Whitney *U* test). **b** Expression of 16 thyroid-specific genes, encompassing the Thyroid Differentiation Score (TDS), in post-mortem tumor tissue of TPO-Cre/LSL-Braf^V600E^ transgenic mice treated with either DMSO vehicle (N = 3), 20 µg digoxin (N = 6) or 60 µg digoxin daily (N = 6). Data are means ± SEM, *P < 0.05 (Mann-Whitney *U* test). **c** Percentage of FOS positive nuclei in post-mortem tumor tissue of TPO-Cre/LSL-Braf^V600E^ transgenic mice treated with either DMSO vehicle (N = 3), 20 µg digoxin (N = 6) or 60 µg digoxin daily (N = 6). **d** Representative immunofluorescent images of nuclear FOS expression in digoxin-treated (left) and vehicle control (right) murine tumors. Scoring results were generated in sextuple for each tissue section. Data are means ± SEM, *P < 0.05 (Mann-Whitney *U* test)

Autophagy activity in mouse thyroid tumors was assessed by LC3 immunofluorescence and expressed as the amount of LC3-II positive puncta per 100 tumor cells. In tumor tissue obtained from either 20 µg or 60 µg digoxin-treated mice about 20 % increased autophagy activity was observed, reaching statistical significance comparing vehicle-treated versus 60 µg digoxin-treated tumors (Fig. 3a). To assess the differentiation status of mouse tumors, mRNA expression of 16 thyroid-specific genes was determined encompassing the previously established Thyroid Differentiation Score (TDS) [34–36]. The overall TDS was increased in digoxin-treated tumors (12.8 mean fold increase after 20 µg digoxin treatment, 8.9 mean fold increase after 60 µg digoxin treatment) as compared to vehicle-treated tumors. Of these 16 genes, especially *Tg*, *Slc5a5* (*Nis*), *Tshr*, *Thra*, *Glis3* and *Tpo* were highly upregulated after digoxin treatment. Furthermore, increased expression of *Pax8* and *Nkx2-1* was observed after treatment with digoxin, although to a lesser extent (Fig. 3b). Previous *in vitro* findings suggested a role for the transcription factor *Fos* in NMTC redifferentiation [16]. Therefore, nuclear *Fos* expression was assessed and profound differences were detected between digoxin-treated and vehicle treated thyroid tumors (Fig. 3c, d).

### Retrospective clinical study

#### Tumors from digoxin-treated NMTC patients exhibit lower proliferative capacity and higher autophagy activity and TDS compared to tumors from matched control NMTC patients

**Fig. 4:**
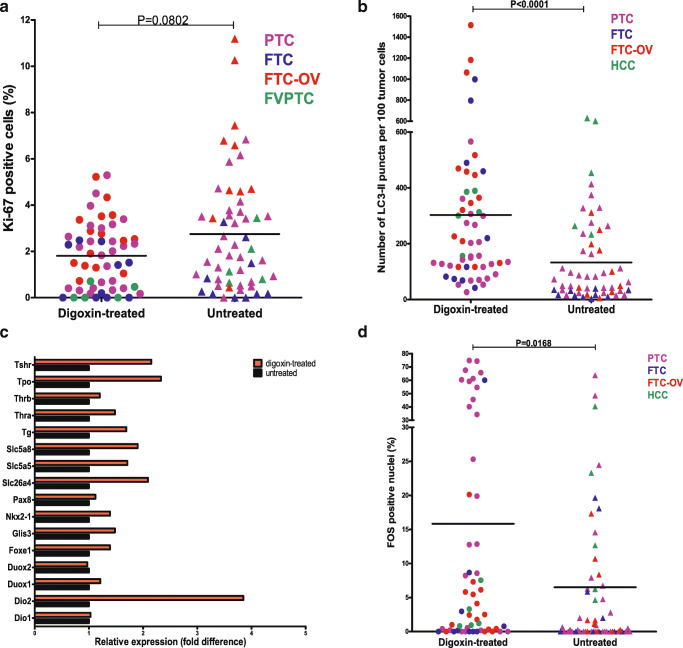
RNA expression profiling, Ki-67 positivity, autophagy activity and nuclear FOS expression in NMTC tumor tissues.** a** Percentage of Ki-67 positive cells in tumor tissues of NMTC patients, stratified for histological tumor subtypes. Scoring results were generated in quintuple for each tissue section; all individual data points are plotted. *P < 0.05 (Mann-Whitney *U* test). **b** Quantification of the number of LC3-II positive puncta per 100 tumor cells. Scoring results were generated in quintuple for each tissue section; all individual data points are plotted. Results were stratified based on histological subtype. *P < 0.05 (Mann-Whitney *U* test). **c** RNA-seq-based expression analysis of thyroid-specific genes in tumor tissues of NMTC patients untreated (N = 11) or treated with digoxin at the time of NMTC diagnosis (N = 11) encompassing all 16 genes incorporated in the Thyroid Differentiation Score (TDS). **d** Percentage of FOS positive nuclei in tumor tissues of NMTC patients, stratified for histological tumor subtypes. Scoring results were generated in quintuple for each tissue section; all individual data points are plotted. *P < 0.05 (Mann-Whitney *U* test). FTC: follicular thyroid cancer; FVPTC: follicular-variant papillary thyroid cancer; HCC: Hürthle cell carcinoma; LC3-II: lipidated form of microtubule-associated protein 1A/1B light chain 3B; NMTC: Non-medullary thyroid cancer; PTC: papillary thyroid cancer;

Through linkage between the Dutch nationwide PALGA and the PHARMO database, including all diagnostic pathology laboratories in The Netherlands and all NMTC cases between 1999 and 2017, 11 NMTC patients could be retrieved that were treated with digoxin at the time of diagnosis and initial treatment of NMTC. Patient and tumor characteristics are listed in Table 1, including age, sex, histology, tumor genetics, TNM stage at diagnosis, prescribed digoxin dose, duration of digoxin treatment and prescribed co-medication. Each digoxin-treated NMTC patient was matched to one appropriate NMTC control patient who was not treated with digoxin and who matched for age, sex, histological tumor type, TNM staging at diagnosis and mutational profile of the tumor. Altogether, 11 digoxin-treated NMTC patients were compared to 11 matched NMTC control patients never treated with digoxin. To determine the proliferative index of digoxin-treated and untreated human NMTC tumor tissues, immunofluorescent Ki-67 staining was performed and quantified. The number of Ki-67 positive cells was not significantly lower in tumor material from digoxin-treated patients as compared to their matched control NMTC patients. Differences in Ki-67 positivity were most apparent in follicular-variant papillary thyroid cancer (FVPTC) and Hürthle cell carcinoma (HCC) subgroups. Assessment of autophagy activity by fluorescent LC3 stainings revealed that tumor tissues of digoxin-treated NMTC patients exhibited an on average 2-fold higher autophagy activity as compared to matched control NMTC patients (Fig. 4a-d). The largest differences in autophagy activity were observed in FTC and HCC histological subgroups. In order to gain insight into differentially expressed genes between tumors obtained from digoxin-treated and untreated NMTC patients, whole transcriptomics analysis was performed by RNA sequencing. High quality RNA sequencing data were obtained for all analysed FFPE tumor tissue specimens ranging from 2.1 to 10^6^ to 5.8 × 10^6^ mapped reads. Subsequently, heatmaps were constructed to visualize expression values of 16 thyroid metabolism- and thyroid function-related genes, together comprising the TDS. TDS was expressed as mean expression value (MEV, average expression value of all TDS genes combined) and mean fold difference (MFD, average expression difference of all TDS genes combined between digoxin-treated patients and matched control patients). Differential expression analysis was performed within the overall comparison of untreated and digoxin-treated NMTC patients and within seven subgroups, categorized based on tumor histology and mutational profile. In all 11 tumor tissues obtained from digoxin-treated NMTC patients the intratumoral differentiation status was higher as compared to matched control NMTC patients with an average TDS fold increase of 1.69 (MEV 29.99 in untreated tumors vs. 49.18 in digoxin-treated tumors, Fig. 4c). Subgroup analysis revealed that in all subgroups digoxin-treated tumors displayed a higher TDS fold difference as compared to their control tumor counterparts, with the highest MFD values observed in FVPTC and HCC subgroups (Fig. S1). Interestingly, also nuclear expression of the transcription factor FOS was significantly higher in tumor tissues from digoxin-treated patients as compared to their untreated counterparts (Fig. 4d).

**Table 1:** Clinical and genetic characteristics of NMTC patients treated with digoxin and matched NMTC patients

Digoxin-treated NMTC patients									Matched NMTC patients not treated with digoxin						
Pt	M/F	Age at diagnosis	TNM staging at diagnosis ^a^	Histology	Mutation status	Digoxin dose (mg daily)	Duration of digoxin treatment before and after NMTC diagnosis	Other medication	**Pt**	M/F	Age at diagnosis	TNM staging at diagnosis*	Histology	Mutation status	Other medication
1	M	88 years	T2N1bM0	PTC	BRAF V600E, TERT C250T	0.0625	5 weeks before, 4 years after	Furosemide	**1–1**	M	82 years	T1bN1bM0	PTC	BRAF V600E, TERT C228T	-
2	M	79 years	T2N0M1	PTC	BRAF V600E, TERT C250T	0.125	14 weeks before, 2 months after	Bisoprolol	**2−1**	M	76 years	T2N1bM1	PTC	BRAF V600E, TERT C228T	-
3	F	88 years	T3N1bM0	PTC	BRAF V600E, TERT C228T	0.125	5 years + 2 months before, 2 years + 7 months after	Furosemide, Bisoprolol	**3−1**	F	84 years	T3N1bM0	PTC	BRAF V600E, TERT C228T	Metoprolol
									**3−2**	F	84 years	T3N1bM0	PTC	BRAF V600E, TERT C228T	Irbesartan
4	F	76 years	T3N1aM1	PTC	BRAF V600E, TERT WT	0.0625 – 0.125	5 weeks before, 2 years + 7 months after	Metoprolol, Perindopril, HCTZ	**4−1**	F	80 years	T3N1bM1	PTC	BRAF V600E, TERT WT	-
5	F	69 years	T1aN1aM0	PTC	BRAF V600E, TERT WT	0.125	4 weeks before, 3 months after	Atenolol	**5−1**	F	69 years	T1bN1aM0	PTC	BRAF V600E, TERT WT	-
6	F	80 years	T3N1aM0	FVPTC	PIK3CA H1047R, TERT C250T	0.125 – 0.25	7 years + 5 months before, 1 year + 7 months after	Furosemide, Bisoprolol, Lisinopril	**6−1**	F	76 years	T3N1aM1	FVPTC	NRAS Q61R, TERT C228T	-
7	F	78 years	T3N1bM0	FTC (PD)	TERT WT	0.25	1 year + 8 months before, 8 years after	Losartan	**7−1**	F	81 years	T4aN0M0	FTC (PD)	KRAS A146T, TERT WT	-
8	F	77 years	T3N1aM1	FTC (WD)	KRAS Q61R, TERT C228T	0.25	2 months before, 2 months after	-	**8−1**	F	82 years	T3N0M1	FTC (WD)	NRAS Q61R, TERT C228T	HCTZ
9	F	81 years	T3N1aM0	HCC	NRAS Q61R, TERT C228T	0.25	5 years + 7 months before, 6 months after	Furosemide, Candesartan	**9−1**	F	74 years	T3N1bM0	HCC	TERT C228T	Insulin
10	F	77 years	T2N1bM0	HCC	PRMT5/PPARG, TERT WT	0.25	6 years + 7 months before, 2 months after	-	**10−1/11−1**	M	72 years	T3N1bM0	HCC	TERT WT	Furosemide, Perindopril, HCTZ, Valsartan, Insulin
11	M	79 years	T3N1aM0	HCC	TERT WT	0.125 – 0.25	10 years + 6 months before, 5 months after	Enalapril							

^a^ Based on AJCC 8th edition of TNM classification

F = female; FTC = follicular thyroid cancer; FVPTC = follicular-variant papillary thyroid cancer; HCC = hürthle cell carcinoma; HCTZ = hydrochlorothiazide; ISMN = isosorbide mononitrate; M = male; NMTC = non-medullary thyroid cancer; PTC = papillary thyroid cancer; PD = poorly differentiated; WD = well differentiated; WT = wild-type

#### Digoxin-treated NMTC patients show a more favourable clinical outcome in comparison to matched control NMTC patients

Comparison of clinical follow-up data of selected digoxin-treated and matched control patients revealed that none of the digoxin-treated patients died from NMTC, whereas five matched control patients died from NMTC-related complications. In addition, three digoxin-treated patients versus nine matched control patients underwent lymph node dissection. Notably, one digoxin-treated patient with distant lung metastases exhibited a complete response to RAI treatment, whereas another digoxin-treated patient displayed stable disease. In contrast, seven matched control patients had proven RAI-resistant tumor lesions and, hence, persistent or recurrent disease. The numbers of RAI treatments and cumulative RAI doses were not significantly different between digoxin-treated patients and matched control patients (Table 2).

**Table 2:** Clinical follow-up of digoxin-treated NMTC patients and matched control NMTC patients

Patient number	TNM stage at diagnosis ^a^	Histology	Lymph node dissection	Residual disease after surgery	Nr. of RAI treatments	Cumulative RAI dose	Disease status after RAI ablation	NMTC-related mortality
Digoxin-treated NMTC patients								
1	T2N1bM0	PTC	No	Yes	1	200 mCi	Remission	No
2	T2N0M1	PTC	No	Yes	1	150 mCi	Remission	No
3	T3N1bM0	PTC	Yes	Yes	2	400 mCi	Remission	No
4	T3N1aM1	PTC	No	Yes	2	400 mCi	Persistent	No
5	T1aN1aM0	PTC	No	Yes	1	150 mCi	Remission	No
6	T3N1aM0	FVPTC	No	Yes	2	400 mCi	Remission	No
7	T3N1bM0	FTC (PD)	Yes	No	1	200 mCi	Remission	No
8	T3N1aM1	FTC (WD)	No	Yes	5	900 mCi	Remission	No
9	T3N1aM0	HCC	No	Yes	2	225 mCi	Remission	No
10	T2N1bM0	HCC	Yes	Yes	3	600 mCi	Remission	No
11	T3N1aM0	HCC	No	Yes	1	200 mCi	Remission	No
NMTC matched control patients not treated with digoxin								
1–1	T1bN1bM0	PTC	Yes	No	1	200 mCi	Remission	No
2−1	T2N1bM1	PTC	Yes	Yes	2	400 mCi	Persistent	Yes
3−1	T3N1bM0	PTC	Yes	Yes	2	400 mCi	Recurrent	No
3−2	T3N1bM0	PTC	No	Yes	1	150 mCi	Remission	No
4−1	T3N1bM1	PTC	Yes	Yes	1	146 mCi	Persistent	No
5−1	T1bN1bM0	PTC	Yes	Yes	1	200 mCi	Remission	No
6−1	T3N1aM1	FVPTC	No	Yes	2	400 mCi	Persistent	Yes
7−1	T4aN0M0	FTC (PD)	Yes	Yes	2	400 mCi	Persistent	No
8−1	T3N0M1	FTC (WD)	Yes	Yes	1	200 mCi	Persistent	Yes
9−1	T3N1bM0	HCC	Yes	Yes	1	200 mCi	Persistent	Yes
10−1/11−1	T3N1bM0	HCC	Yes	Yes	2	400 mCi	Recurrent	Yes

^a^ Based on AJCC 8th edition of TNM classification

FTC = follicular thyroid cancer; FVPTC = follicular-variant papillary thyroid cancer; HCC = hürthle cell carcinoma; NMTC = non-medullary thyroid cancer; PTC = papillary thyroid cancer; PD = poorly differentiated; RAI = radioactive iodide; WD = well differentiated

## Discussion

Although most NMTC patients have a favourable prognosis, a substantial subgroup develops advanced, metastatic disease that is often accompanied by dedifferentiation and RAI resistance. Since treatment options are limited, this frequently leads to persistent disease and increased risk of NMTC-related mortality. Restoring RAI uptake by reactivating thyroid-specific gene expression, designated as redifferentiation, is considered a promising therapeutic approach for RAI-refractory NMTC [2, 3]. The present study supports the therapeutic potential of digoxin to induce redifferentiation and to increase RAI uptake in NMTC *in vivo*.

The cardiac glycoside digoxin is a well characterized Na^+^/K^+^ ATPase inhibitor that has been used for treatment of heart disease for many decades by virtue of its capacity to enhance cardiomyocyte contractility by increasing intracellular Ca^2+^ concentrations with minimal side effects [37, 38]. Digoxin is considered as potential anticancer therapy because of the pleiotropic downstream effects of intracellular Ca^2+^ modulation influencing multiple cancer-related pathways, including proliferation, apoptosis, autophagy and differentiation [39–41]. In NMTC cells, digoxin has been found to reactivate thyroid-specific gene expression and to restore iodide uptake with activation of autophagy and the transcription factor FOS as potential underlying mechanisms [16, 17]. The current study shows the *in vivo* effects of digoxin treatment on NMTC tumor growth and RAI uptake capacity. In TPO-Cre/LSL-Braf^V600E^ transgenic mice diminished tumor proliferation and increased iodide avidity was observed in thyroid tumors after a short course of digoxin treatment, accompanied by a reduced Ki-67 proliferation index, increased autophagy activity and enhanced thyroid-specific gene expression. Of note, no dose-response effects were observed in this study between 20 µg and 60 µg dosages, indicating that administration of 20 µg digoxin is sufficient to reach the maximal effect in mice. This could indicate that lower dosages of digoxin might be equally effective which, however, remains to be demonstrated.

In the retrospective human study, profound differences in transcriptomic tumor profiling, characterized by markedly higher thyroid-specific gene expression in digoxin-treated tumors, were observed. Differences in thyroid-specific gene expression were detected in all histological subgroups with higher TDS fold differences for all digoxin-treated NMTC patients. In addition, Ki-67 and LC3 immunofluorescent staining revealed profoundly lower proliferation indexes and significantly higher autophagy activities in digoxin-treated NMTC patients. To exclude that our findings are influenced by known confounding factors, digoxin-treated NMTC patients were closely matched with control NMTC patients for age, sex, histological tumor type, TNM staging at diagnosis and mutational profile. Despite the relative small sizes of the selected digoxin-treated and matched control NMTC patient cohorts, statistically significant differences in autophagy activity and thyroid-specific gene expression were observed. Moreover, we found that these data are in line with data obtained from *in vivo* TPO-Cre/LSL-Braf^V600E^ mouse experiments. Of note, the effects of digoxin treatment observed in the *in vivo* mouse model were more pronounced compared to the retrospective human study. These differences might be explained by differences in digoxin dose and by the fact that all mice developed poorly differentiated tumors, whereas the patient cohort represents a more heterogeneous group.

Interestingly, digoxin-treated NMTC patients were more prone to reach remission after RAI treatment, suggesting that digoxin treatment could be associated with a more favourable clinical outcome. These observations are in agreement with the molecular findings of activated autophagy, reduced proliferation and enhanced thyroid-specific gene expression reported here. In addition, these effects could be explained by other potentially involved anti-cancer mechanisms known to be activated by digoxin, including enhancement of immunogenic cell death, sensitization to anoikis, increased production of reactive oxygen species and inhibition of (pseudo)hypoxia, angiogenesis, glycolysis and epithelial-mesenchymal transition [42–47].

In the present study, the dose of digoxin administered to mice was higher than the dose range applied for the treatment of heart disease in humans. However, it has been shown that the presence of the α1 subunit of the murine Na^+^/K^+^ ATPase, which binds digoxin poorly, renders murine cells less sensitive to the digoxin-mediated effects and that higher doses of digoxin are required *in vivo* to elicit these effects [48, 49]. The observational data from our human cohort of digoxin-treated NMTC patients support the hypothesis that the effects observed in our animal model could be elicited in humans using therapeutic doses of digoxin since these tumors also exhibited higher autophagy activity, lower proliferative capacity, higher TDS and favourable clinical outcomes compared to matched control NMTC patients not treated with digoxin. Therefore, when designing future proof of principle human studies, the known maximum tolerated dose of digoxin should be used.

The transcription factor FOS has previously been identified as a crucial mediator of digoxin-induced reactivation of thyroid-specific gene expression *in vitro*, especially of the sodium iodide symporter (SLC5A5, NIS), but also involving calmodulin, ERK and cAMP signalling and CREB phosphorylation [16, 17, 50, 51]. Strikingly, in both mouse and in human tumors profound differences in nuclear FOS expression have been observed after digoxin treatment. In mouse tumors, the number of tumor cells with FOS-positive nuclei was evidently increased after digoxin treatment. Accordingly, we found that tumor tissues obtained from digoxin-treated NMTC patients exhibited higher nuclear FOS expression than their matched control NMTC patients. These data suggest that digoxin-induced NMTC redifferentiation, especially reactivation of NIS expression, is at least partially mediated by FOS both *in vitro* and *in vivo*, presumably by binding to the NIS upstream enhancer in cooperation with PAX8, as described previously [52].

In the past decade, several multi-kinase inhibitors have been shown to improve the progression-free survival of patients with RAI-refractory NMTC [53]. Currently, sorafenib and lenvatinib are approved for treatment of RAI-refractory thyroid cancer patients [9, 54]. Targeted kinase therapies have also been previously assessed for their redifferentiation potential in NMTC, including the MEK inhibitor selumetinib and the BRAF^V600E^ inhibitors vemurafenib and dabrafenib [55–57]. The clinical efficacy of digoxin as compared to that of the abovementioned multi-kinase inhibitors remains to be investigated in future clinical trials. Importantly, clinical application of digoxin in patients with heart disease is characterized by a more favourable safety and toxicity profile as compared to these targeted kinase therapies in cancer patients [9, 53, 54, 58]. These profiles apply when digoxin or targeted kinase therapies are administered separately. However, one must be aware of drug-drug interactions when combinations of these drugs are administered in clinical practice. In a recent review, Akbulut et al. raised awareness of possible drug-drug interactions between cardiovascular drugs and anti-cancer drugs [59].

A few limitations of this study should be mentioned. The retrospective design of the human study and the small number of patients require future validation in prospective studies with larger sample sizes. Different digoxin dosages were used in mice and patients. However, the patients did receive the therapeutic dose of digoxin, which apparently was effective. Also, the use of a single assay to evaluate LC3 in order to detect autophagy activity is a limitation. Therefore, future studies are needed to confirm our findings from both the retrospective cohort as well as the mouse study.

Taken together, we here report the *in vivo* effects of digoxin treatment on NMTC proliferation, redifferentiation and RAI uptake capacity, both in mice and in humans. These findings need validation in prospective human studies and may potentially establish a repositioned clinical application of the cardiac glycoside digoxin as adjunctive treatment modality to suppress tumor growth and to improve RAI responsiveness in RAI-refractory NMTC patients.

## Supplementary Information

ESM 1 (DOCX 173 kb)

## Abbreviations

ATC: anatomical therapeutic chemical
cDNA: complementary DNA
DAPI: 4’,6-diamidino-2-phenylindole
DLC: digitalis-like compound
FFPE: formalin-fixed paraffin embedded
FTC: follicular thyroid cancer
FVPTC: follicular-variant papillary thyroid cancer
HCC: hürthle cell carcinoma
MEV: mean expression value
MFD: mean fold difference
NIS: sodium-iodide symporter
NMTC: non-medullary thyroid cancer
PET/CT: positron emission tomography / computer tomography
PTC: papillary thyroid cancer
RAI: radioactive iodide
smMIP: single-molecule molecular inversion probe
TDS: thyroid differentiation score
TG: thyroglobulin
TPO: thyroid peroxidase
TSHR: thyroid stimulating hormone receptor
