# Supplementary material for: Digoxin treatment reactivates in vivo radioactive iodide uptake and correlates with favorable clinical outcome in non‐medullary thyroid cancer
Source: Cell Oncol (Dordr). 2021 Feb 3;44(3):611–25. doi: 10.1007/s13402-021-00588-y (PMC8213564; doi:10.1007/s13402-021-00588-y)
Supplement: Supplementary file 1 — (DOCX 173 kb) [file 13402_2021_588_MOESM1_ESM.docx]

**Supplementary information**

**Supplementary Table 1.**

Primer sequences for real-time quantitative PCR on cDNA obtained from post-mortem Tpo-Cre/LSL-Braf^V600E^ mouse tumor tissues.

| Gene | 5’🡪 3’ forward primer | 5’🡪 3’ reverse primer |
| --- | --- | --- |
| Dio1 | GCT-GAA-GCG-GCT-TGT-GAT-ATT | GTT-GTC-AGG-GGC-GAA-TCG-G |
| Dio2 | AAT-TAT-GCC-TCG-GAG-AAG-ACC-G | GGC-AGT-TGC-CTA-GTG-AAA-GGT |
| Duox1 | AAA-ACA-CCA-GGA-ACG-GAT-TGT | AGA-AGA-CAT-TGG-GCT-GTA-GGG |
| Duox2 | AAG-TTC-AAG-CAG-TAC-AAG-CGA-T | TAG-GCA-CGG-TCT-GCA-AAC-AG |
| Foxe1 (Ttf2) | ATC-GCG-CTC-ATC-GCT-ATG-G | GGG-GTT-GTC-GCG-GTA-GAA-C |
| Glis3 | TGT-GGC-ATG-AAT-CTC-CAC-CG | AGG-TGA-AGA-CTG-TTA-GCA-AGA-CT |
| Nkx2-1 (Ttf1) | AGG-ACA-CCA-TGC-GGA-ACA-G | CCA-TGC-CGC-TCA-TAT-TCA-TGC |
| Pax8 | ATG-CCT-CAC-AAC-TCG-ATC-AGA | ACA-ATG-CGT-TGA-CGT-ACA-ACT-T |
| Slc26a4 | CAG-CGG-TAA-CGG-AAG-TGC-AT | TCT-GCC-AAG-TAC-CTC-ACT-ATG-A |
| Slc5a5 (NIS) | CGC-TAC-GGT-CTC-AAG-TTT-CTG | CGC-AGT-TCT-AGG-TAC-TGG-TAG-G |
| Slc5a8 | TTA-TGG-GCG-GTC-GCA-GTA-TG | CAA-AAC-GGT-AGA-CCT-CGG-CA |
| Tg | CTG-GTC-TTG-TGG-GTC-TCT-ACT | CAC-TGG-GGA-ACA-TAT-TCA-GCC |
| Thra | GGT-CAC-CAG-ATG-GAA-AGC-GAA | CCT-TGT-CCC-CAC-ACA-CGA-C |
| Thrb | ACA-CCA-GCA-ATT-ACC-AGA-GTG | GCA-GCT-CGA-AGG-GAC-ATG-A |
| Tpo | CTT-GGA-GCT-ATG-GCA-ATA-ATG-CT | GAG-CTG-GCT-CGT-TTC-CAC-A |
| Tshr | CAC-CAG-GAG-GAC-GAC-TTC-AGA | GGC-AGA-CTC-GAA-AAT-GCA-AGA |
| B2m (housekeeping gene) | TTC-TGG-TGC-TTG-TCT-CAC-TGA | CAG-TAT-CTT-CGG-CTT-CCC-ATT-C |

**
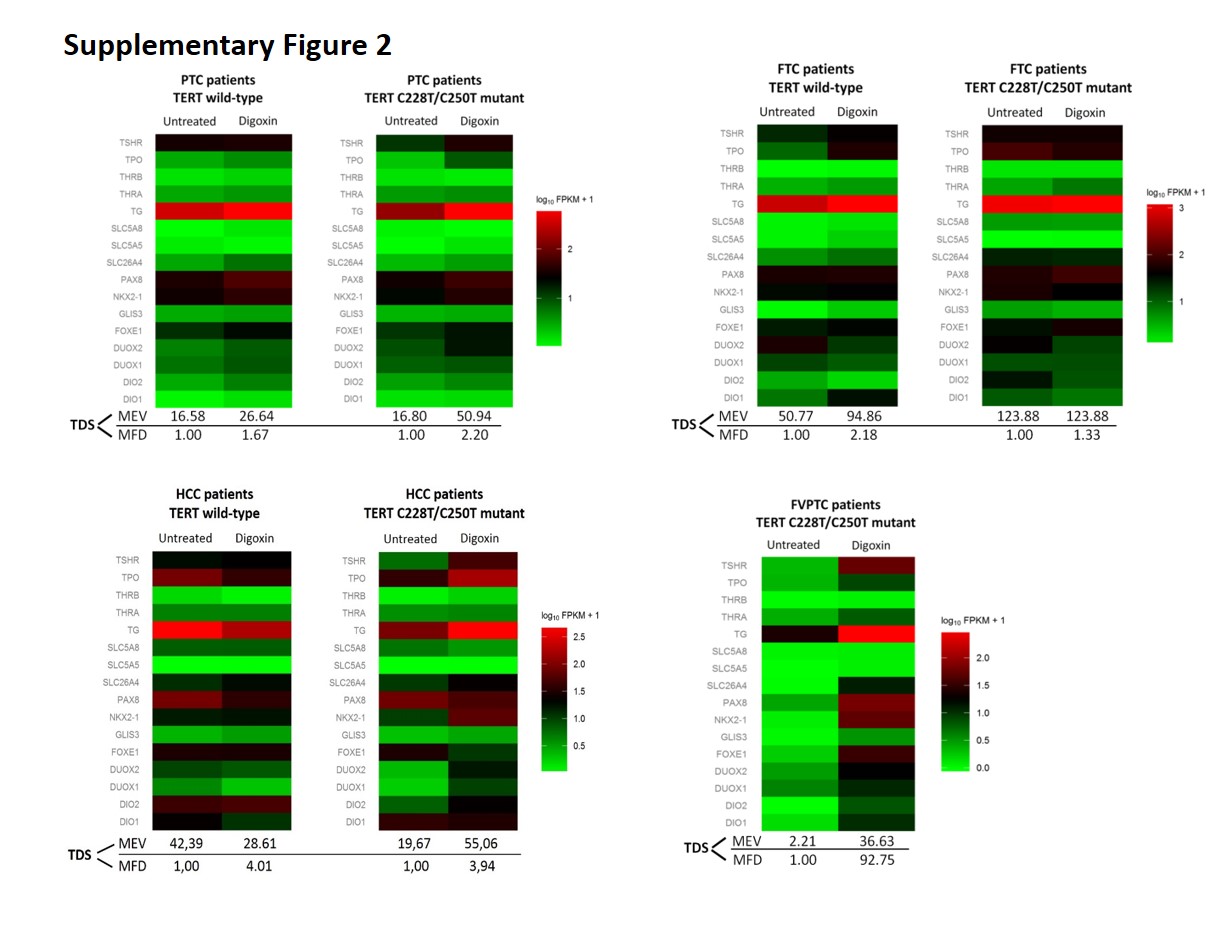
**

**Supplementary Figure 1**

**Supplementary Fig 1** Heatmaps of thyroid-specific gene expression in NMTC tumor tissue after histological and genetic stratification. MEV: mean expression value of all 16 TDS genes; MFV: mean fold difference of all 16 TDS genes between untreated NMTC patients (reference value, 1.00 by default) and NMTC patients treated with digoxin. Subgroups represent:

(1) PTC patients with TERT wild-type tumor (digoxin treated, N=2; untreated matched control, N=2);
(2) PTC patients with TERT mutant tumor (digoxin treated, N=3; untreated matched control, N=4);
(3) FTC patients with TERT wild-type tumor (digoxin treated, N=1; untreated matched control, N=1); (4) FTC patients with TERT mutant tumor (digoxin treated, N=1; untreated matched control, N=1);
(5) HCC patients with TERT wild-type tumor (digoxin treated, N=2; untreated matched control, N=1);
(6) HCC patients with TERT mutant tumor (digoxin treated, N=1; untreated matched control, N=1);

(7) FVPTC patients with TERT mutant tumor (digoxin treated, N=1; untreated matched control, N=1). FTC: follicular thyroid cancer; FVPTC: follicular-variant papillary thyroid cancer; HCC: Hürthle cell carcinoma; MEV: Mean Expression Value; MFD: Mean Fold Difference; PTC: papillary thyroid cancer; TDS: Thyroid Differentiation Score.
